# scHLAcount: allele-specific HLA expression from single-cell gene expression data

**DOI:** 10.1093/bioinformatics/btaa264

**Published:** 2020-04-24

**Authors:** Charlotte A Darby, Michael J T Stubbington, Patrick J Marks, Álvaro Martínez Barrio, Ian T Fiddes

**Affiliations:** b1 Department of Computer Science, Johns Hopkins University, Baltimore, MD 21218, USA; b2 10x Genomics, Pleasanton, CA 94588, USA

## Abstract

**Summary:**

Bulk RNA sequencing studies have demonstrated that human leukocyte antigen (HLA) genes may be expressed in a cell type-specific and allele-specific fashion. Single-cell gene expression assays have the potential to further resolve these expression patterns, but currently available methods do not perform allele-specific quantification at the molecule level. Here, we present scHLAcount, a post-processing workflow for single-cell RNA-seq data that computes allele-specific molecule counts of the HLA genes based on a personalized reference constructed from the sample’s HLA genotypes.

**Availability and implementation:**

scHLAcount is available under the MIT license at https://github.com/10XGenomics/scHLAcount.

**Supplementary information:**

Supplementary data are available at *Bioinformatics* online.

## 1 Introduction

The class I and class II human leukocyte antigen (HLA) genes play an important role in antigen presentation in the immune system, and are highly variable in the human population with hundreds of cataloged alleles (Robinson *et al.*, 2015). Studies using bulk RNA-seq data have shown that HLA genes are expressed at different levels among human tissues and immune cell types (Boegel *et al.*, 2018), and allele-specific expression (ASE) has been observed in lymphoblastoid cell lines (Aguiar *et al.*, 2019; Lee *et al.*, 2018). However, expression of these genes may be underestimated in RNA-seq experiments due to poor read mapping caused by sequence divergence between the standard reference genome and the alleles in the reads.

It is particularly useful to understand ASE of HLA genes in the context of single cells and particular cell types. For example, cell type-specific HLA class I and class II expression can influence immunotherapy response in cancer (Chowell *et al.*, 2018; Johnson *et al.*, 2016). scHLAcount enables allele-specific analysis of the HLA genes in single-cell gene expression data, such as those produced by the 10× Genomics Single Cell Immune Profiling (5′ capture) and Gene Expression (GEX) (3′ capture) Solutions. Based on the genotypes of the sample, scHLAcount constructs a personalized reference and computes allele-specific molecule counts for HLA class I and class II genes. This output can be used to study ASE of HLA genes at the single-cell resolution.

## 2 Implementation

**Fig. 1.**
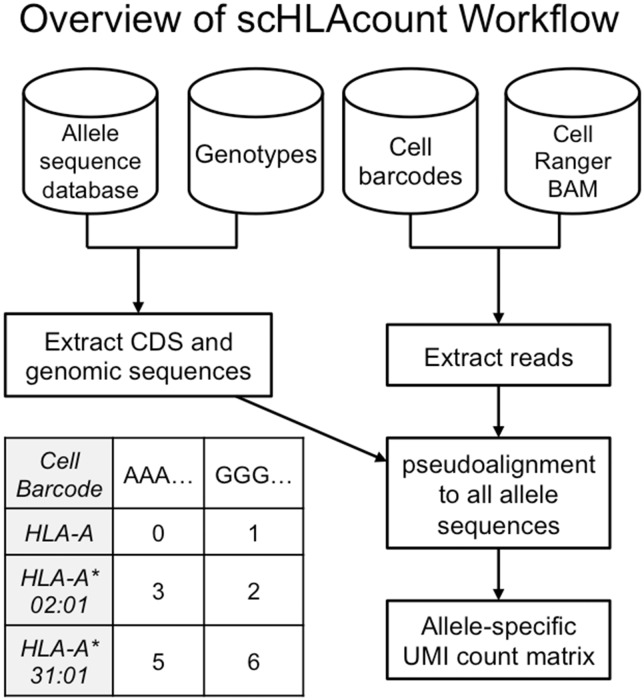
scHLAcount takes as input an allele sequence database (e.g. IMGT/HLA), genotypes for the sample being evaluated, cell barcodes and aligned reads (e.g. BAM file from Cell Ranger). Allele sequences and relevant reads are extracted, and pseudoalignment is used to produce an allele-specific molecule count matrix. A snippet of the output matrix is shown for two cell barcodes and one gene (HLA-A) with two alleles

scHLAcount is a post-processing workflow for single-cell gene expression data that produce allele-specific molecule counts for the main HLA class I and class II genes in each cell (Fig. 1). Users provide the specific HLA alleles present in their sample of interest. These can be obtained by specialized molecular tests, such as sequence-specific oligonucleotide probe PCR, sequence-specific primed PCR, or Sanger sequence-based typing (Erlich, 2012). Alternatively, algorithms for sequence-based typing from next-generation sequencing reads of the genome, exome or transcriptome that use allele databases to infer genotypes can be employed [reviewed by Bauer *et al.* (2018)]. Tian *et al.* (2019) attempted to genotype individual cells for HLA class I using scRNA-seq data, but found that most cells did not have adequate read coverage. Combining reads from many cells in an scRNA-seq experiment as a ‘pseudo-bulk’ dataset for genotyping is an interesting avenue for further research.

Based on the genotypes provided, scHLAcount extracts the coding and genomic sequences of those alleles from the IMGT/HLA database (Robinson *et al.*, 2015) and builds two colored de Bruijn graphs, one containing the coding sequences (CDS) and one containing genomic sequences. In addition, scHLAcount uses the read alignments generated by scRNA-seq analysis tools such as Cell Ranger. Reads associated with valid cell barcodes and reported as aligning to the region of the genome containing the HLA genes are extracted from the alignment file and pseudoaligned to the CDS graph following the approach described by Bray *et al.* (2016). This yields the set of alleles in the reference graph that could have generated the read, also referred to as the equivalence class. If there is no significant alignment to the CDS graph, pseudoalignment is attempted to the genomic sequence graph. In 5′ GEX datasets, we observed up to 12% of aligned reads were only aligned to the genomic sequence graph and not the CDS graph. In 3′ GEX datasets, up to 80% of aligned reads were aligned to the genomic sequence. This genomic alignment step is intended to rescue reads that may be haplotype specific in 3′ or 5′ untranslated regions (UTR). It also provides a mechanism to handle reads from pre-mRNA in single nuclei RNA-seq libraries. Parameters and approaches to missing genotypes are discussed in Supplementary Material S1.

Reads sharing a cell barcode and unique molecular identifier (UMI) are assumed to originate from the same RNA molecule. At recommended sequencing depths with modest sequence saturation, there are typically 1–3 reads per UMI. Individual reads may have different equivalence classes according to their pseudoalignment. We ignore reads whose equivalence class contains more than one gene, which we observed was 15–45% of aligned reads in 5′ GEX datasets and 10% of reads in 3′ GEX. If more than half of the reads from a molecule are assigned to a particular gene, that molecule will be assigned to one of its input reference alleles (e.g. HLA-A 02:01), based on the constituent reads’ equivalence classes. In the case of ambiguity, it will be assigned to that gene (e.g. HLA-A) instead. The output is a sparse molecule count matrix where each column corresponds to a barcode in the provided cell barcode list, and each row corresponds to an allele. See Supplementary Material S2 for a more detailed comparison of 3′ and 5′ GEX data with scHLAcount.

## 3 Results

To illustrate the applications of scHLAcount, we reanalyzed two previously published datasets. First, we applied our method to five acute myeloid leukemia (AML) samples (Petti *et al.*, 2019) (Supplementary Material S3). Using the scHLAcount allele-specific molecule counts, we detected cell type-specific allele bias. Detailed results from one patient are shown in Supplementary Figure S2 and Tables S1–S3. Second, we reexamined data from two Merkel cell carcinoma (MCC) patients (Paulson *et al.*, 2018) (Supplementary Material S4). We extend the original finding that HLA class I expression is lost in tumor cells compared with non-tumor cells and use scHLAcount allele-specific molecule counts to show that this expression loss may be allele-specific (Supplementary Fig. S3 and Tables S4–S6).

## 4 Conclusion

scHLAcount provides a simple way to assign reads from scRNA-seq experiments to HLA alleles given genotypes, and is a powerful tool for investigating ASE, loss of heterozygosity and mutational or epigenetic suppression of HLA expression in tumor immune-evasion.

## Supplementary Material

btaa264_Supplementary_Data
