# Supplementary material for: scHLAcount: allele-specific HLA expression from single-cell gene expression data
Source: Bioinformatics. 2020 Apr 24;36(12):3905–6. doi: 10.1093/bioinformatics/btaa264 (PMC7320622; doi:10.1093/bioinformatics/btaa264)
Supplement: btaa264_Supplementary_Data [file btaa264_supplementary_data.pdf]

Supplementary Materials for scHLAcount: Allele-specific HLA expression from single-cell gene expression data

Charlotte A. Darby, Michael J. T. Stubbington, Patrick J. Marks, Álvaro Martínez Barrio, Ian T. Fiddes

April 8, 2020

Supplementary Note 1: Parameter Considerations

Parameter selection For the experiments described here, we used the following parameter settings, which are customizable by users of our tool:

- k-mer length of 20 for de Bruijn graph
- minimum pseudoalignment length of 60 bases
- maximum 2 mismatches in pseudoalignment

These parameters were selected based on our test datasets with genotypes with two or three-field resolution, where we expect the personalized reference to have very few mismatches with the allele present in the reads. scHLAcount selects an arbitrary allele from the database consistent with the provided genotypes. If the genotypes provided are lower-resolution (e.g. the one-field genotype A*02 is lower-resolution than the three-field genotype A*02:01:01), scHLAcount arbitrarily selects a representative sequence from all A*02 alleles. Therefore, when only lower-resolution genotypes are available, the pseudoalignments of reads to the personalized reference may contain more mismatches and users may want to decrease the k-mer length or decrease the minimum significant alignment length.

Missing Genotypes If genotypes for any of the seven genes currently analyzed by scHLAcount are not available, the genotype of the GRCh38 reference should be used for consistency with the initial reference genome based read alignment step. Because this information does not appear to be available in the literature, we performed a simple simulation-based analysis of the GRCh38 primary assembly using Kourami v0.9.6 (Lee and Kingsford, 2018) to determine which alleles were present. 2 million 200bp error-free reads were simulated from GRCh38 Chr6:28510120-33480577, which is approximately 80-fold coverage of the region. Reads were aligned to the Kourami reference panel and genotypes were inferred; all listed genotypes had 100% sequence identity with respect to the corresponding database sequence. The GRCh38 genotypes are A*03:01:01G, B*07:02:01G, C*07:02:01G, DQA1*01:02:01G, DQB1*06:02:01G, DRB1*15:01:01G, DPA1*01:03:01G, DPB1*04:01:01G.

Supplementary Note 2: 3' GEX data versus 5' GEX data

Due to the nature of 3' GEX data, nearly all reads are sequenced from the opposite end of the HLA-A transcript from the variable sites used to define HLA types (Supplementary Figure 1). These variable sites are mostly located in exons 2 and 3, while the 3' end of the transcripts are mostly homologous between the class I genes (Boegel *et al.*, 2018). As a result of the coverage distribution of 3' GEX data, very few HLA-A molecules could be assigned to an allele in the two 3' GEX samples in Supplementary Table 4. This is because UMIs that only contain reads from the 3' end of the transcript (UMIs typically contain 1-3 reads, so this is not uncommon) will be pseudoaligned to an equivalence class including both HLA-A alleles. Comparing the 3' GEX molecule assignment percentages (Supplementary Table 4) to the 5' GEX results (Supplementary Tables 1 and 4) indicates that 5' GEX data is preferable to 3' GEX data for assigning molecules to alleles, because the sequencing coverage is not as limited to one end of the transcript.

Supplementary Tables 1 and 4 also compare the total (pre-normalization) scHLAcount molecule counts to the molecule counts from Cell Ranger for the HLA genes with genotypes available. Depending on the gene, assay type (5' GEX versus 3' GEX), and sample, we sometimes observe more molecules from scHLAcount and sometimes more from Cell Ranger. We consistently observe fewer molecules from scHLAcount in 3' GEX data, possibly due to read distribution along the transcript (Supplementary Figure 1) and the fact that most reads must be pseudoaligned to the genomic sequence graph (see Implementation). Total molecule count could also be affected by the sequence similarity of the specific alleles in the sample or expression level of the HLA genes in the sample.

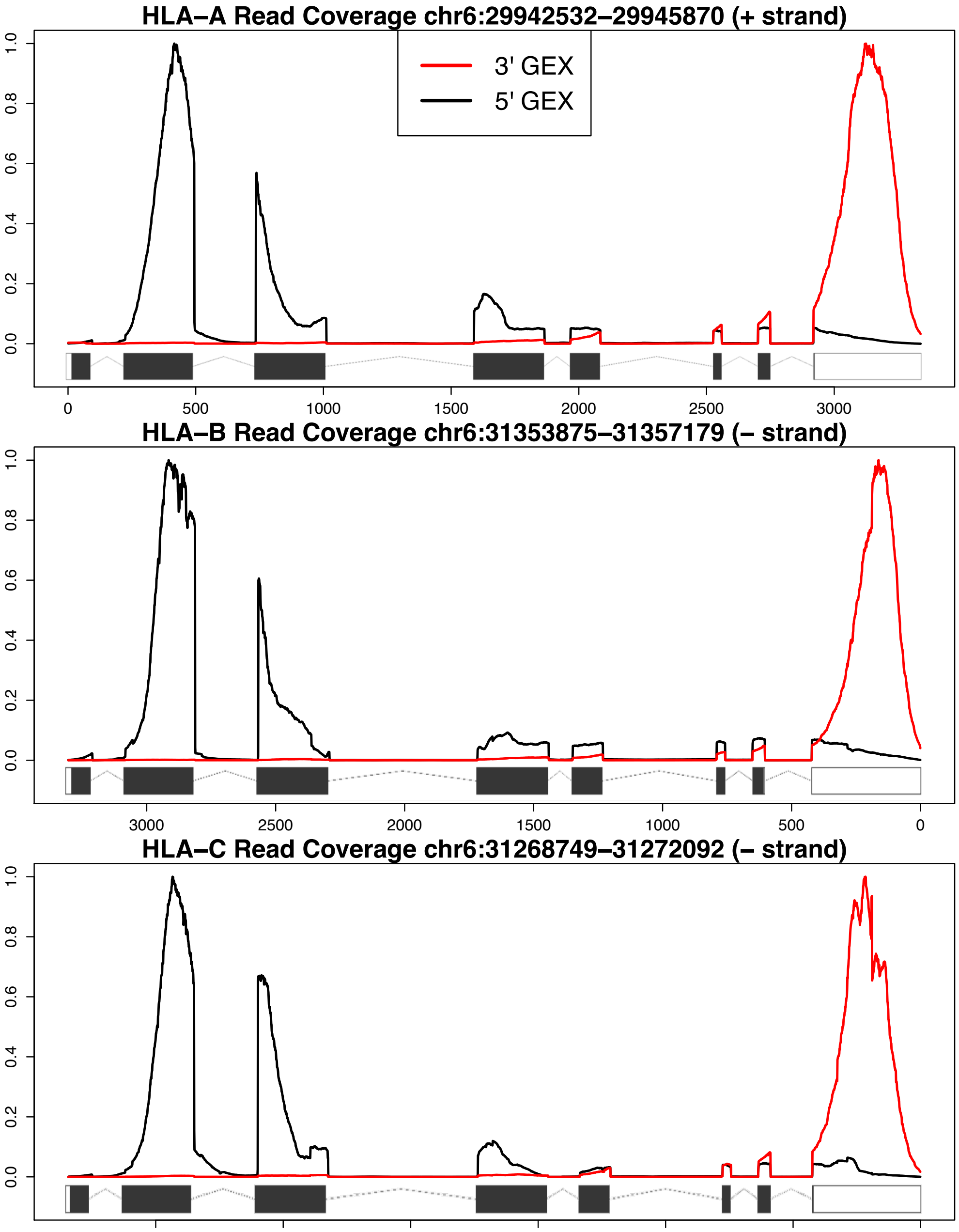

Supplementary Figure 1: Read coverage of HLA Class I genes for 3' GEX and 5' GEX. Minimum and maximum coverage for each assay in the region shown is normalized to 0 and 1 respectively. The value in thousands of reads for the maximum coverage for genes A, B, C is (3'/5') 47/192, 97/288, 68/286. The 3' dataset is merged from SRR7722937-SRR7722942 and the 5' dataset is SRR7692286, all from Paulson *et al.* (2018). GEX = gene expression

Supplementary Note 3: Acute myeloid leukemia (AML)

Data 10x Genomics Chromium 5' GEX library data derived from five subjects with AML, as described in (Petti *et al.*, 2019) was reanalyzed. Genotypes for HLA-A, -B, -C, -DRB1, and -DQB1 at two-field resolution were provided by the authors. As described in Supplementary Note 1, reference genotypes were used for HLA-DQA1, -DPA1, and -DPB1 since these genotypes were unavailable. scHLAcount genotype files are available at `https://github.com/10XGenomics/scHLAcount/tree/master/paper`.

Analysis Analysis scripts are available at `https://github.com/10XGenomics/scHLAcount/tree/master/paper`. Raw scHLAcount molecule counts are summarized in Supplementary Table 1. Molecule counts were then normalized with the following formula:

$$\text{median molecule count} \times \text{raw molecule count}/\text{cell molecule count}$$

Normalization and dimensionality reduction of the gene expression matrix generated by Cell Ranger v2.1.1 was performed using Seurat v3.0.2 (Stuart *et al.*, 2019). For all the biallelic genes in each subject, we calculated the average normalized expression per gene and the fraction of the normalized expression for each allele of the nine cell types with at least 100 cells assigned.

Results Some genes had more expression of one allele than the other. Results for subject 809653 with the class II gene HLA-DRB1 are listed in Supplementary Table 2 and visualized on a t-SNE dimensionality reduction plot in Supplementary Figure 2a,b. Depending on cell type, we observe 42% to 54% allelic bias for the DRB*01:03 allele. This allele preference does not show a trend with average expression. For the same subject, we also observe a 27% to 41% allelic bias for C*07:02 depending on cell type (Supplementary Figure 2c,d; Supplementary Table 3).

Subject	Custom diploid reference	% molecules assigned to an allele	Custom diploid reference	% molecules assigned to an allele	Custom diploid reference	% molecules assigned to an allele	Custom diploid reference	% molecules assigned to an allele
	HLA-A		HLA-B		HLA-C		HLA-DQB1	
508084	1.039	95.13	1.066	87.22	0.885	60.77	1.028	95.89
548327	1.165	86.26	1.061	93.09	1.032	n/a	2.721	2.27
721214	1.180	69.44	1.137	90.09	0.908	93.63	3.319	98.95
782328	1.154	n/a	0.880	63.95	0.957	89.77	1.010	99.15
809653	1.083	87.21	1.154	96.53	0.911	91.74	1.070	n/a

Subject	Custom diploid reference	% molecules assigned to an allele	GRCh38 allele	GRCh38 allele	GRCh38 allele
	HLA-DRB1		HLA-DPA1	HLA-DPB1	HLA-DQA1
508084	1.641	74.60	1.135	1.024	1.086
548327	1.920	89.52	1.180	1.172	2.087
721214	1.745	89.05	1.217	1.050	2.058
782328	1.125	92.12	1.276	1.078	1.274
809653	1.066	95.43	1.136	1.050	1.455

Supplementary Table 1: Using the custom diploid reference or GRCh38 allele as denoted, raw molecule count for each gene is compared to Cell Ranger counts normalized to 1.0. Subject 548327 is homozygous for HLA-C, Subject 782328 is homozygous for HLA-A, and Subject 809653 is homozygous for HLA-DQB1. (Related to Supplementary Note 3)

Cell type	# cells	% of DRB1 molecules assigned to 01:03 allele	% of DRB1 molecules assigned to 11:01 allele	Avg. HLA-DRB1 normalized expression
ERY	3,728	41.9	58.1	0.238
T-CELL	10,942	44.8	55.2	0.741
PRE-B-CELL	336	47.4	52.6	1.162
B-CELL	868	47.4	52.6	14.185
HSC	2,261	52.1	47.9	5.247
MEP	560	53.0	47.0	3.411
DEND (M)	620	53.7	46.3	17.602
ERY (CD34+)	432	53.9	46.1	2.153
MONO	1,366	54.0	46.0	7.390

Supplementary Table 2: Normalized expression and allele-specific expression of HLA-DRB1 for subject 809653 from (Petti *et al.*, 2019), stratified by cell type. Average is taken over all cells assigned to a particular cell type. (Related to Supplementary Note 3)

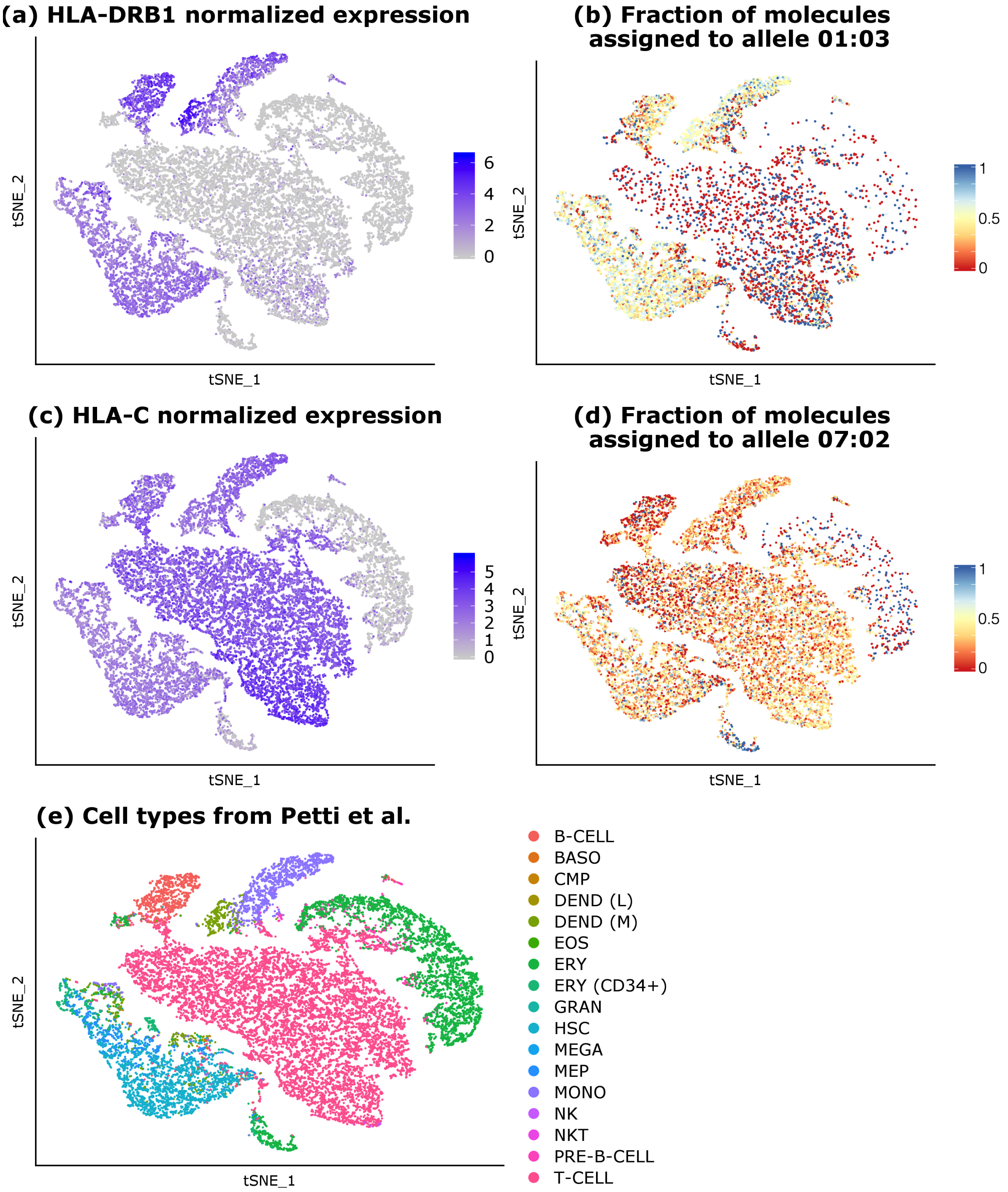

Supplementary Figure 2: **(a)** For each cell, color indicates $\log_2(1 + \text{normalized expression})$ of HLA-DRB1. **(b)** For each cell, color indicates the fraction of HLA-DRB1 molecules assigned to an allele that are assigned to the 01:03 allele of subject 809653. Overall, 95.4% of HLA-DRB1 molecules are assigned to an allele. Gray cells have no HLA-DRB1 molecules assigned to an allele. **(c)** $\log_2(1 + \text{normalized expression})$ of HLA-C **(d)** **(e)** Cell types as inferred in (Petti *et al.*, 2019). (Related to Supplementary Note 3)

Cell type	# cells	% of HLA-C molecules assigned to 07:02 allele	% of HLA-C molecules assigned to 08:02 allele	Avg. HLA-C normalized expression
B-CELL	868	26.7	73.3	5.184
MONO	1,366	32.7	67.3	5.813
PRE-B-CELL	336	33.9	66.1	3.266
DEND (M)	620	35.1	64.9	3.890
T-CELL	10,942	37.0	63.0	8.926
HSC	2,261	38.8	61.2	3.281
MEP	560	40.3	59.7	2.578
ERY (CD34+)	432	40.9	59.1	2.429
ERY	3,728	41.0	59.0	0.386

Supplementary Table 3: Normalized expression and allele-specific expression of HLA-C for subject 809653 from (Petti *et al.*, 2019), stratified by cell type. Average is taken over all cells assigned to a particular cell type. (Related to Supplementary Note 3)

Supplementary Note 4: Merkel cell carcinoma (MCC)

Data Genotypes for genes HLA-A, -B, and -C for the discovery and validation subjects in (Paulson *et al.*, 2018) were provided to us by the authors. Here, alleles not explicitly reported in their publication are given a placeholder name (e.g. A1/A2) for confidentiality. Using scHLAcount with a custom reference for the diploid genotype of genes HLA-A, -B, and -C (and GRCh38 primary assembly alleles for the class II genes as described in Supplementary Note 1) we calculated allele-resolved molecule counts. Raw molecule counts were normalized as described above. For the discovery subject, we used the filtered expression matrices for tumor and PBMC samples available at GEO accession GSE117988; for the validation subject, the matrix is available at GSE118056.

Analysis Analysis scripts are available at `https://github.com/10XGenomics/scHLAcount/tree/master/paper`. Normalization, dimensionality reduction, and clustering was performed using Seurat v3.0.2 (Stuart *et al.*, 2019) following Paulson et al (Paulson *et al.*, 2018).

Analysis and Results: Discovery subject For this subject, the "tumor dataset" comprises cells taken from two time points in treatment; the "PBMC dataset" comprises cells taken from four time points in treatment. Unsupervised clustering of the tumor dataset resulted in 15 clusters. As described in Paulson et al (Paulson *et al.*, 2018), we identified 11 of these clusters comprising 7,131 cells as putative tumor cells using the tumor marker genes NCAM1, KRT20, CHGA, and ENO2 and the non-tumor marker genes CD3D, CD34, CD61, and Fibronectin. The remaining four clusters contained 300 putative normal cells.

As previously reported, HLA-B expression is markedly less in the tumor compared to non-tumor cells and PBMC (Supplementary Table 5). Additionally, HLA-A and HLA-C expression appears to be reduced in tumor cells.

Analysis and Results: Validation subject For this subject, the "tumor dataset" and "PBMC dataset" comprise cells taken from a single time point after relapse. Unsupervised clustering of all cells together resulted in 18 clusters. As described in Paulson et al (Paulson *et al.*, 2018), we identified seven of these clusters comprising 4,682 cells as putative tumor cells using the tumor marker genes NCAM1, KRT20, Large T Antigen, and Small T Antigen. Only 17 of these cells originated from the PBMC dataset. The remaining 6,209 cells were designated putative normal cells and comprised 5,731 cells from the PBMC dataset and 478 cells from the tumor dataset, which (Paulson *et al.*, 2018) identified as tumor-infiltrating leukocytes and tumor-associated macrophages (Supplementary Figure 2e). Compared to Cell Ranger molecule counts, we inferred more molecules for the PBMC dataset and fewer molecules for the tumor dataset. At least 80% of scHLAcount molecules were assigned to an allele for class I genes (Supplementary Table 4).

Dividing cells into tumor and normal as described above, we corroborate the observation from (Paulson *et al.*, 2018) that HLA-A expression is greatly reduced in tumor cells compared to infiltrating immune cells (Supplementary Figure 3a). No marked allele-specific bias in expression is observed in cells in either category. Additionally, we observe decreased expression of HLA-B and HLA-C in tumor cells (Supplementary Figure 3c,e). While non-tumor cells display approximately balanced expression of the two alleles of these genes, tumor cells have only 13% of allele-resolved HLA-B expression from allele 35:01 and 6% of allele-resolved HLA-C expression from allele 'C1' (Supplementary Table 6).

Subject	Assay type	Custom diploid reference	% molecules assigned to an allele	Custom diploid reference	% molecules assigned to an allele	Custom diploid reference	% molecules assigned to an allele
		HLA-A		HLA-B		HLA-C	
Discovery (Tumor)	3' GEX	0.866	5.34	0.391	40.76	0.639	64.31
Discovery (PBMC)	3' GEX	0.855	6.42	0.449	45.98	0.767	67.94
Validation (Tumor)	5' GEX	0.878	81.17	0.896	91.69	0.745	80.68
Validation (PBMC)	5' GEX	1.050	87.71	1.073	94.41	1.033	89.65

Supplementary Table 4: scHLAcount analysis of discovery patient tumor (2 time points) and PBMC (4 time points) and validation patient tumor and PBMC (1 time point each) (Paulson *et al.*, 2018). Raw molecule counts for genes A, B, and C are compared to Cell Ranger counts normalized to 1.0. GEX = gene expression (Related to Supplementary Note 4)

Gene Genotype	Tumor cells (n=7,131)		Non-tumor cells (n=300)		PBMC (n=12,874)	
	Average normalized expression	% molecules assigned to alleles	Average normalized expression	% molecules assigned to alleles	Average normalized expression	% molecules assigned to alleles
HLA-A A1/A2	0.724	24.98/75.02	3.392	43.78/56.22	1.958	40.83/59.17
HLA-B 35:02/B2	0.115	76.11/23.89	3.156	61.70/38.30	1.713	63.97/36.03
HLA-C C1/C2	0.209	49.54/50.46	3.802	59.58/40.42	1.918	59.17/40.83

Supplementary Table 5: Average overall and allele-specific expression of HLA class I genes in the discovery subject of (Paulson *et al.*, 2018). (Related to Supplementary Note 4)

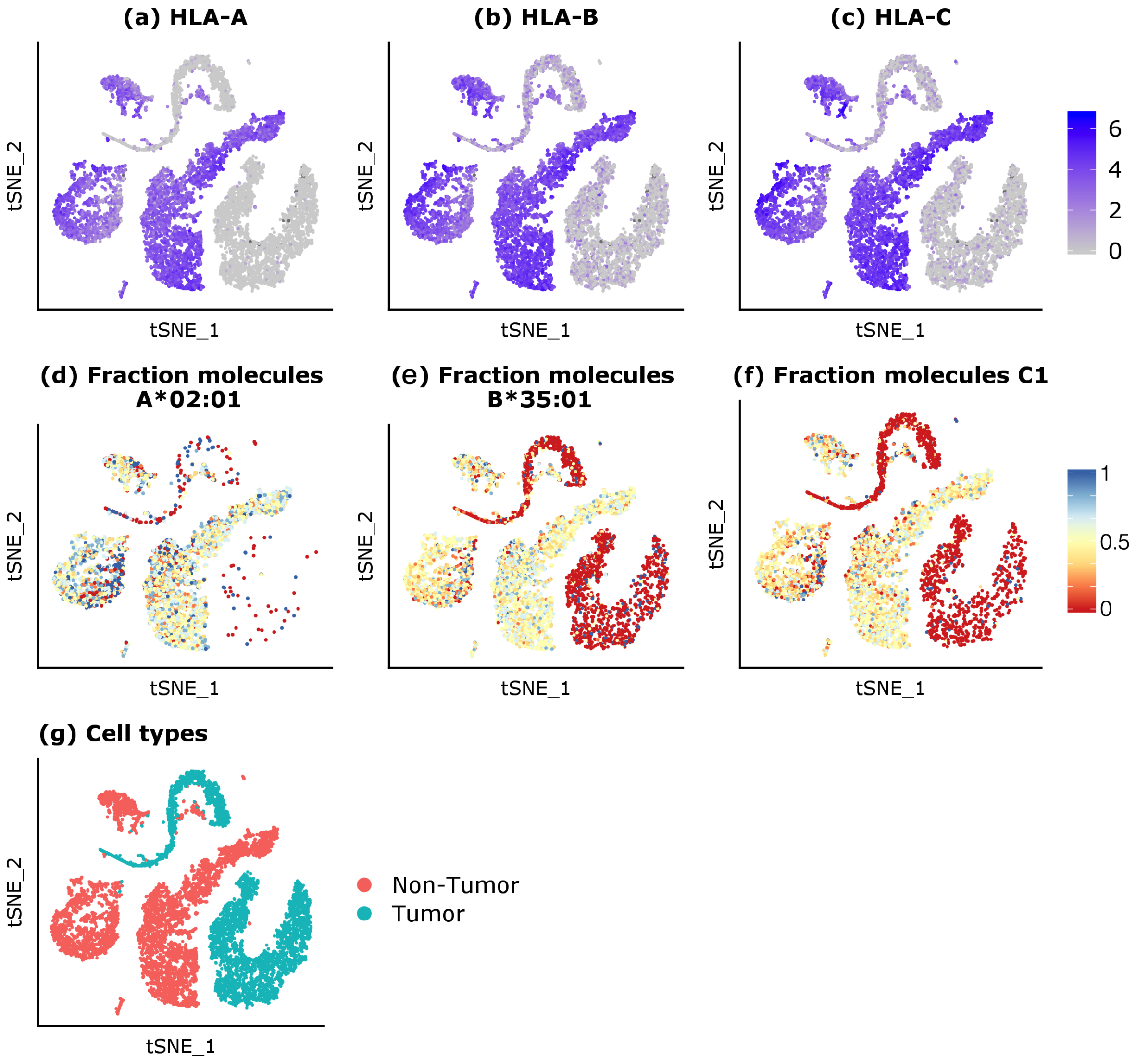

Supplementary Figure 3: $\log_2(1 + \text{normalized expression})$ of HLA-A (a) HLA-B (b) and HLA-C (c) and allele preference for HLA-A*02:01 (d) HLA-B*35:01 (e) and HLA-C1 (f) for the validation subject of (Paulson *et al.*, 2018). Values are plotted per cell; aggregate statistics shown in Supplementary Table 6. (g) Cell types inferred using marker genes. (Related to Supplementary Note 3)

Gene<br>Genotype	Tumor cells<br>(n=4862)		Non-tumor cells<br>(n=6209)	
	Average normalized expression	% molecules assigned to allele 1	Average normalized expression	% molecules assigned to allele 1
HLA-A<br>02:01/A2	0.060	39.7/60.3	4.154	56.8/43.2
HLA-B<br>35:01/B2	0.511	13.4/86.6	5.172	50.4/49.6
HLA-C<br>C1/C2	0.327	6.3/93.7	4.991	46.8/53.2

Supplementary Table 6: Average overall and allele-specific expression of HLA class I genes in the validation subject of (Paulson *et al.*, 2018). (Related to Supplementary Note 4)

References

Boegel, S. *et al.* (2018). HLA and proteasome expression body map. *BMC Medical Genomics*, **11**(1), 36.

Lee, H. and Kingsford, C. (2018). Kourami: graph-guided assembly for novel human leukocyte antigen allele discovery. *Genome Biology*, **19**(1), 16.

Paulson, K. G. *et al.* (2018). Acquired cancer resistance to combination immunotherapy from transcriptional loss of class I HLA. *Nature Communications*, **9**(1), 3868.

Petti, A. A. *et al.* (2019). A general approach for detecting expressed mutations in AML cells using single cell RNA-sequencing. *Nature Communications*, **10**(1), 3660.

Stuart, T. *et al.* (2019). Comprehensive Integration of Single-Cell Data. *Cell*, **177**(7), 1888–1902.e21.
